# New Application of Electricity to Surgery

**Published:** 1845-07

**Authors:** 


*New application of Electricity to Surgery.*—A novel application of electricity has been described by Mr. Smee at his lectures on Surgery, delivered at the Aldersgate Street School of Medicine. He states that needles and other steel instruments are frequently impacted in the human body, and do irretrievable mischief, but may be detected by rendering them magnetic. The following extracts will sufficiently explain the *modus operandi*:—

"You are all acquainted with the curious condition which steel assumes under certain circumstances, whereby it evinces properties which are called magnetic; you know, moreover, that like magnetic poles repel, and opposite attract each other. You have, therefore, but to render a piece of enclosed steel a magnet, and you will be able not only to ascertain its presence, but to determine by its polarity its general direction; and by the amount of magnetism it evinces, you may even infer its probable bulk.

"When you suspect the presence of a piece of needle, or other steel instrument, you must subject the suspected part to a treatment calculated to render the needle magnetic; and there are two principal methods by which this object may be effected;—the first, by transmitting a galvanic current, at right angels, to the suspected part; the second, by placing a large magnet near the part affected, so that the object may be magnetized by induction. You may accomplish the first end, by taking a copper wire, covered with cotton, or still better with silk (in fact, you may employ the covered wire as generally used for the formation of electro-magnets,) and wind it round the parts suspected to contain steel, several times, so that the same current may act at right angles, many times, upon the piece of steel; you may then take a galvanic battery (one of my little tumbler batteries will amply suffice,) and connect one end of the wire to the zinc, the other to the platinized silver. The current might be continued for half an hour, or more, when the steel would become magnetized, and thereby give strong indications of its presence.

"For my own part, I should use the second plan, or the plan of magnetizing by induction, to render the needle magnetic. For this purpose I have employed a temporary electro-magnet, which I magnetized by the voltaic battery, and you will find, that by keeping the part affected as close as possible to the instrument for about half an hour, you will sufficiently obtain the desired object.

"The electro-magnet might be made of the horse-shoe form, if we knew the direction of the object; but in that case we should not require its use at all, as the proof of the existence of the needle is our only aim. I have used the horse-shoe magnet, but should prefer, in most cases, an electro-magnet like this, made for me by Messrs. Horne of Newgate Street, which is made of a simple straight bar of soft iron, wound round with wire. Your chemical lecturer has, doubtlessly, made you aware that the magnetic effect is proportionate to the power of the battery, so that if you were desirous of producing but slight effect, you would employ this tumbler battery; but if you require the action to be manifested to a greater distance, you would use a compound battery, such as this trough battery upon the table. The compound battery will magnetize a needle, in conjunction with the electro-magnet, in the space of two or three minutes. A powerful permanent magnet would answer as well as the temporary magnet; but it would be very expensive, and not so constantly at hand. When soft iron is impacted in any part of the body, we do not require either the electro or permanent magnet, for on this substance we are unable to confer magnetic properties.

"To test the existence of a magnet within the body, we may take a magnetized sewing needle, and suspend it by a piece of sikworm's silk, when it will exhibit certain phenomena upon the approach of the subjected part, provided it contain a piece of magnetized steel. Although this simple contrivance will amply suffice, I myself possess a needle which was made for me by Messrs. Willats of Cheapside, and which is well adapted for the purpose.

"It consists, as you will perceive, of a delicate needle, about six inches long, centered upon a small agate cup, resting upon a steel point; so that the smallest possible amount of resistance is offered to its free play.

"When a part containing magnetic steel is brought near the needle, it may be either attracted or repelled, it may move upwards or downwards, or it may exhibit disquietude according to the position in which the new magnet is held. We may detect the position of the foreign body, when it is of any size, by ascertaining where its north and south poles lie, and these are determined by their repelling and attracting the opposite poles of the magnetic needle. The disquietude, or motion upwards and downwards, merely indicates magnetism, but not the direction of the magnet.

"You will, doubtless, be surprised when I tell you, that in this manner I have detected a piece of needle impacted in the finger of a young woman, although it weighed but the seventh of a grain. This gave such marked indications, that I found out tolerably well the positions of its north and south poles, though I could not ascertain the presence of a foreign body in any other way. I tried experiments on smaller pieces, at shorter distances, such as half an inch to an inch, and I found that a piece of needle, weighing 1-60th of a grain, gave decided indications after having been magnetized, and perhaps even a still smaller amount of steel might in some cases be detected.

"I have now satisfactorily demonstrated to you, that magnetism may be used for the detection of steel particles impacted within the body with absolute success; and though but a very trifling application of natural philosophy to the practice of surgery, I have no doubt that, had it been adopted before, many joints would have been saved; and I confidently anticipate that it will be the means, in future, of frequently saving these parts from destruction."—*Lecture on the Detection of Needles and other Steel Instruments.*

